# Caregivers in implantable brain-computer interface research: a scoping review

**DOI:** 10.3389/fnhum.2024.1490066

**Published:** 2024-10-31

**Authors:** Nicolai Wohns, Natalie Dorfman, Eran Klein

**Affiliations:** ^1^Department of Philosophy, University of Washington, Seattle, WA, United States; ^2^Oregon Health and Science University, Portland, OR, United States

**Keywords:** caregivers, brain-computer interface, neuroethics, bioethics, neuroscience

## Abstract

**Introduction:**

While the ethical significance of caregivers in neurological research has increasingly been recognized, the role of caregivers in brain-computer interface (BCI) research has received relatively less attention.

**Objectives:**

This report investigates the extent to which caregivers are mentioned in publications describing implantable BCI (iBCI) research for individuals with motor dysfunction, communication impairment, and blindness.

**Methods:**

The scoping review was conducted in June 2024 using the PubMed and Web of Science bibliographic databases. The articles were systematically searched using query terms for caregivers, family members, and guardians, and the results were quantitatively and qualitatively analyzed.

**Results:**

Our search yielded 315 unique studies, 78 of which were included in this scoping review. Thirty-four (43.6%) of the 78 articles mentioned the study participant’s caregivers. We sorted these into 5 categories: Twenty-two (64.7%) of the 34 articles thanked caregivers in the acknowledgement section, 6 (17.6%) articles described the caregiver’s role with regard to the consent process, 12 (35.3%) described the caregiver’s role in the technical maintenance and upkeep of the BCI system or in other procedural aspects of the study, 9 (26.5%) discussed how the BCI enhanced participant communication and goal-directed behavior with the help of a caregiver, and 3 (8.8%) articles included general comments that did not fit into the other categories but still related to the importance of caregivers in the lives of the research participants.

**Discussion:**

Caregivers were mentioned in less than half of BCI studies in this review. The studies that offered more robust discussions of caregivers provide valuable insight into the integral role that caregivers play in supporting the study participants and the research process. Attention to the role of caregivers in successful BCI research studies can help guide the responsible development of future BCI study protocols.

## 1 Introduction

In recent years, increasing attention has been given to the significance of caregivers in neurological research, most notably in research on Alzheimer’s disease and on deep-brain stimulation (DBS). Multiple ethical issues have been discussed. One is the contribution that caregivers and family members make to decision-making, especially in the context of cognitive impairment (Largent et al., 2022). Another relates to the levels of support that caregivers provide to study participants, to researchers, and to the success of a clinical trial. These can be wide-ranging and include such activities as engaging in research tasks at home and in clinical settings (Gulley et al., 2021; Mao et al., 2018; Pais-Vieira and Garcia, 2019) and monitoring the study participant’s cognitive function and emotional well-being during the trial (Scharre et al., 2018; Boulicault et al., 2023; Thomson et al., 2023). In light of these findings, some scholars have argued that caregivers should be integrated more formally into study protocols in neuroscientific research such as those for Alzheimer’s disease (Grill and Karlawish, 2017).

In addition to elucidating the ways in which caregivers provide support in clinical trials, scholarly work has focused on how clinical trials affect caregivers themselves. The physical and emotional burden of caregiving in the context of conditions such as Alzheimer’s disease, Parkinson’s disease, and ALS has been a particular focus (Haahr et al., 2013; Haahr et al., 2020). For instance, one study documents how DBS for Alzheimer’s disease alters caregiver-patient relationships (Viaña and Gilbert, 2019). Other research has developed burden scales and reported on qualitative interviews with caregivers, investigating the extent to which they were affected by their participation in clinical trials (Mao et al., 2018; Reuben et al., 2019). This work has been important in expanding scholarship on caregiver involvement in these research contexts.

There has been less scholarship, however, on the role of caregivers in other fields of neurological research outside of Alzheimer’s disease and Parkinson’s disease, such as research in the use and development of implantable brain-computer interfaces (BCIs). This is perhaps surprising, for a large part of research in this area involves individuals with significant motor impairment who often rely on caregivers to perform activities of daily living. Yet, lately there has been growing interest in the subject. For example, recent work proposes a standard methodology for research into the home use of communication BCIs and argues for the importance of integrating caregivers into research protocols (Vansteensel et al., 2022). For many participants with significant disabilities, communication can be complicated. Establishing, minimally, a way of communicating yes/no answers to questions is required, and caregivers who know participants well may be best placed to help assess best communication strategies. Vansteensel and colleagues rightfully emphasize that the practical and emotional support provided by caregivers is essential to the success of research into the home use of BCIs.

While we wholeheartedly agree with the above methodological recommendations and believe they are important to advance the field, it is not yet clear how many BCI studies involve caregivers, and more empirical work needs to be done to understand the current nature of caregiver involvement in BCI research. To our knowledge, there have been no systematic assessments of caregiver involvement in BCI studies. To begin this process, we performed a scoping review to identify and catalog the ways in which caregivers are mentioned in published studies of implantable BCIs. We defined the scope of our review to include BCI research for paralysis, aphasia, and blindness, as these conditions are currently the major experimental applications for BCIs (Miller et al., 2020). Understanding how caregivers are discussed in BCI research publications is not only important to honor the important work that they do and respect the integral role they play in the lives of the participants, but is also a step towards developing a comprehensive methodology that responsibly integrates caregivers in future trials.

## 2 Materials and methods

The scoping review was conducted in June 2024 using the PubMed and Web of Science bibliographic databases. Guidelines given by the Preferred Reporting Items for Systematic Reviews and Meta-Analyses (PRISMA) were followed throughout, and the review protocol was published with the Open Science Framework. To identify the keywords for our search, we looked at the keywords used in previous reviews of BCI research (Livanis et al., 2024; Miller et al., 2020), and iteratively refined them to best capture all relevant implantable BCI research studies for motor dysfunction, communication impairment (caused by stroke, ALS, or spinal cord injury), and blindness. This process culminated in the query terms found in Table 1.

**Table 1:** Search queries.

Database	Search query
Pubmed	((((brain-computer) OR (brain-machine)) OR (neuroprosthesis) AND (humans[Filter])) AND ((((intracortical) OR (intracortically)) OR (microelectrode array)) OR (electrocorticography) AND (humans[Filter]))) AND ((((((((((stroke) OR (paralysis)) OR (amyotrophic)) OR (motor neuron disease)) OR (visual)) OR (vision)) OR (blind)) OR (blindness)) OR (communication)) OR (speech) AND (humans[Filter])).
Web of Science	(((TS = (brain-computer) OR TS = (brain-machine)) OR TS = (neuroprosthesis)) AND TS = (human) AND (((TS = (intracortical) OR TS = (intracortically)) OR TS = (microelectrode array)) OR TS = (electrocorticography))) AND (((((((((TS = (stroke) OR TS = (paralysis)) OR TS = (amyotrophic)) OR TS = (motor neuron disease)) OR TS = (visual)) OR TS = (vision)) OR TS = (blind)) OR TS = (blindness)) OR TS = (communication)) OR TS = (speech)).

The articles were imported to Rayyan, a tool that allows efficient deduplication and screening. Rayyan gives each article a percentage score of the words and characters that are similar to other articles. The articles that score high (e.g., >80%) are reviewed, and if the titles are identical to other articles in the search, one of the duplicates is removed. The following inclusion criteria were then applied: studies involving implantable BCIs, involving human study participants with stroke, cervical spinal cord injury, locked-in syndrome, ALS, or visual disability, and articles with full text available in English. We excluded articles that involved BCIs that were not surgically implanted or were exclusively focused on non-human animals, in a non-English language, or for which the full text was not available. We also excluded articles that were not clinical studies of BCIs, such as review articles, articles involving previously collected data reporting new, purely technical innovations, e.g., new deep-learning algorithms, that did not involve direct contact with research participants. Articles that involved post-hoc analysis of clinical data from implantable BCIs were included only if they included a report of the clinical details of the participants. We also excluded studies that exclusively involved patients with epilepsy (cf. Verwoert et al., 2022) or intraoperative ECoG recordings during brain tumor surgery (*cf.* Delfino et al., 2021).

**Figure 1:**
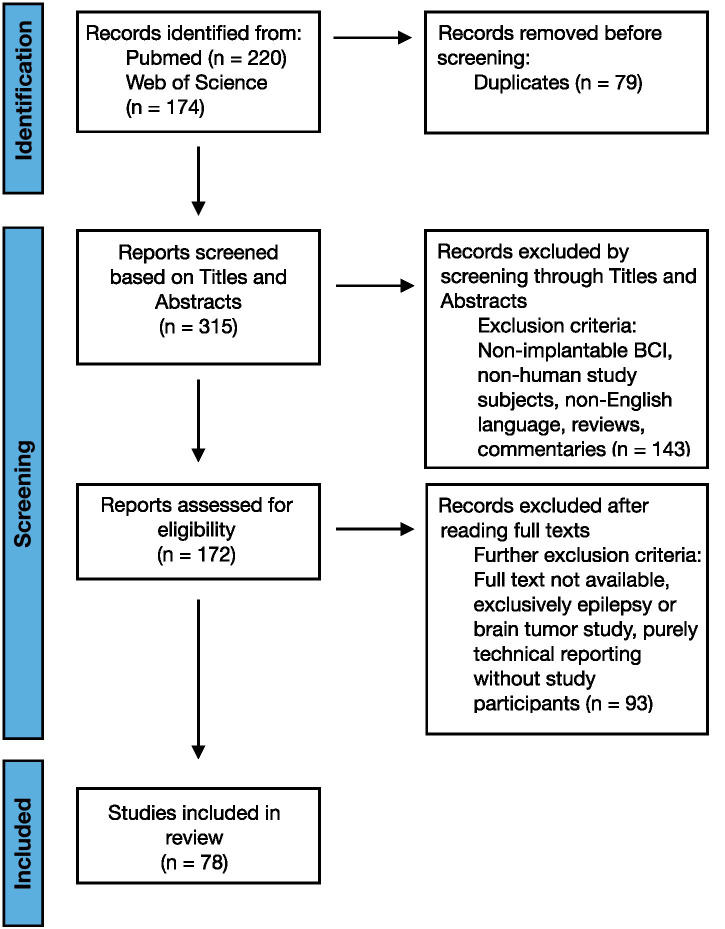
Systematic review PRISMA flowchart demonstrating the steps of searching, screening, and selecting data. PRISMA: Preferred Reporting Items for Systematic Reviews and Meta-Analyses.

The screening process is shown in Figure 1. The search resulted in a total of 394 papers (220 studies on PubMed and 174 on Web of Science) that met the query criteria, limited to articles from 2004 through June 2024. There were 79 duplicate articles between the two databases, and their removal resulted in 315 unique articles. In the first screening phase, articles were reviewed by title and abstract by each reviewer separately (NW and ND), resulting in the exclusion of 143 articles. The full text of the remaining articles were reviewed, and articles were excluded on the basis of the exclusion criteria. Screening conflicts were resolved through discussion between the two reviewers. This process resulted in 78 articles being included in the review.

We systematically searched the articles for discussion of caregivers, using the following search terms: caregiver, caretaker, care partner, support partner, study partner, family, spouse, husband, wife, guardian, parent, child. Since the review is focused on the dyad of study participant and their caring other and the role of the dyad in research, whether the ‘caring other’ is a family member or a non-family-member caregiver, we chose query terms to reflect the fact that the caring other can be represented by a diverse range of individuals, from a family member to a hired caretaker to a legally appointed guardian. While acknowledging that caregivers are sometimes not family members and vice versa, we often use ‘caregiver’ and ‘family member’ interchangeably in this review, mostly preferring the term “caregiver” for expediency. For the remaining extraction step, the following entities were recorded from the articles included in this scoping review: the type of underlying medical condition of study participants, number of study participants, function of BCI being studied, and whether or not the included article discussed caregivers (or related terms). The two reviewers, NW and ND, worked in parallel for data extraction. Divergent decisions were resolved through discussion between the two reviewers and through independent review of the full text of the article in question.

The primary analysis consisted of a qualitative assessment of the relationship between BCI trials and the involvement of caregivers in the trial. The secondary analysis involved further qualitative assessment of relationships among the independent variables and the dependent variable. Synthesis was assessed and reviewed by other members of the University Neuroethics Research Group. Divergent synthesis decisions were adjudicated by the co-authors through in-depth discussion of the data and relevant research details, as well as consulting the University Neuroethics Research Group for critical feedback.

For a quantitative analysis of the comments regarding caregivers, we used a 1-way ANOVA test to estimate how word count changes according to the category of comment, given one independent variable (category of comments regarding caregivers) and one quantitative dependent variable (word count). Five categories were used, as detailed in Appendix B. The null hypothesis of the ANOVA was that there was no difference in word count among categories. We performed a post-hoc Tukey test to specify the pairs of data that cause the difference in means identified from the ANOVA.

## 3 Results

### 3.1 Study characteristics

Forty-seven of the 78 studies (60%) had only one research participant (Appendix A). The remaining 32 (40%) studies had two or more participants: 16 (50%) of these studies involved heterogenous medical conditions (e.g., one participant with motor neuron disease and one with spinal cord injury). The motor and communication impairments of the study participants had a variety of causes. Spinal cord injury was the most common condition in the review, represented in 35.9% of the studies (28 of 78). ALS was represented in 19.2% (15 of 78), stroke in 21.8% (17 of 78), locked-in syndrome in 10.3% (8 of 78), upper extremity amputation in 3.8% (3 of 78), spinocerebellar degeneration in 2.6% (2 of 78), blindness in 2.6% (2 of 78), and essential tremor in 1.3% (1 of 78). Nine (11.5%) of the 78 studies involved at least one patient with tetraplegia without mentioning a specific cause.

The function of the BCIs differed among the studies, with some of the studies investigating multiple functions within the same study (Table 2). BCIs in 7 (9.0%) of the articles focused on speech, such as decoding the neural activity associated with attempted speech, either by itself, combined with “brain-to-text” generation, or reproducing spoken language. BCIs in 32 (41%) of the articles were used to control a cursor on a computer, of which 7 were specifically described as used for typing. An additional study (1.3%) of BCI-mediated typing did not rely on cursor control. Twenty-two (28.2%) of the articles involved BCIs for upper extremity movement, either the participant’s own paralyzed limb (10 studies), a prosthetic limb (5 studies), a virtual limb (5 studies), or a robotic limb (5 studies). One BCI controlled both the participant’s paralyzed limb and a robotic limb, and another BCI controlled both the participant’s paralyzed limb and a virtual limb. Four (5.1%) of the studies reported on sensory feedback strategies: 3 involving BCI-mediated microstimulation of the somatosensory cortex and 1 involving skin-shear haptic stimulation of the back of the neck, as a substitute for proprioception, to improve cursor-control performance. Two (2.6%) articles involved BCIs for research into visual processes. Nine (11.5%) articles reported only on different aspects of motor imagery decoding without specifying functions that would fall into other categories as listed here. One (1.3%) article reported on a BCI used to control a flight simulator.

**Table 2:** Study characteristics.

Variable	Number of studies (% of total)
Sample size	
1 study participant	47 (60%)
2–6 participants	27 (35%)
>6 participants	4 (5%)
Medical condition	
Spinal cord injury	28 (35.9%)
ALS	15 (19.2%)
Stroke	17 (21.8%)
Upper extremity amputation	3 (3.8%)
Spinocerebellar degeneration	2 (2.6%)
Blindness	2 (2.6%)
Essential tremor	1 (1.3%)
Locked-in syndrome (without specific cause mentioned)	8 (10.3%)
Tetraplegia (without specific cause mentioned)	9 (11.5%)
BCI function	
Speech-related	7 (9%)
Cursor control	32 (41%)
Limb movement	22 (28.2%)
Sensory feedback	4 (5.1%)
Vision-related	2 (2.6%)
Motor imagery decoding only	9 (11.5%)
Flight simulator	1 (1.3%)

See Appendix A for further study details.

### 3.2 Mention of caregivers

Of the 78 studies in the review, 34 (43.6%) articles included at least one mention of caregivers. We first separated these into two major categories: (1) statements about caregivers in general, and (2) observations of caregivers in the specific research trial being reported (see Appendix B for the categorization and examples). Eight of the 34 studies (23.5%) that mentioned caregivers included at least one statement belonging to the first category. These were comments about caregivers in general, for instance mentioning them in a hypothesized or idealized way. For example, the Introduction in Moses et al. (2021) includes the following: “Anarthria hinders communication with family, friends, and caregivers, thereby reducing patient-reported quality of life.” Similarly, the Introduction of Simeral et al. (2011) reads, “Most individuals with tetraplegia depend on caregivers for mobility and physical interaction with their environment.” And finally, Rubin et al. (2023) write, “People with quadriparesis may have greater contact with caregivers attending to health-related needs than those with epilepsy or Parkinson disease; consequently, comparisons with devices intended for ambulatory populations are imperfect.”

The remaining comments regarding caregivers, those belonging to category 2, were specific reports of the observed role of caregivers in the study. We categorized this group thematically into four sub-categories: (2a) thanking the caregivers involved with the study, (2b) describing the caregiver’s role with regard to the study’s consent process, (2c) describing the caregiver’s role in the technical maintenance and upkeep of the BCI apparatus or in other procedural aspects of the study, and (2d) discussing the ways in which the BCI enhanced communication and goal-directed behavior with the help of a caregiver.

Instances of category 2a, where caregivers were thanked in the acknowledgement section of the study, were found in 22 (64.7%) of the 34 articles. The following is an example: “We thank participant T12 and her caregivers for their generously volunteered time and effort as part of the BrainGate2 pilot clinical trial” (Willett et al., 2023). The content and form of samples in this category did not vary significantly (further examples can be found in Appendix B). For category 2b, there were 6 (17.6%) articles that described the caregiver’s role with regard to the study’s consent process. Caregivers, guardians, and family members helped provide consent for entry into the research study or for surgical implantation of the BCI. Referring to a study participant with tetraplegia caused by a spinal cord injury, Jiang et al. (2022) write, “Informed consent was obtained from FL and his family for this implantable BCI study aiming to achieve real-time neural signal decoding and subsequent control of a high-performance prosthetic limb.” Similarly, in a study with a participant with locked-in syndrome, Chaudhary et al. (2019) write, “The legally responsible family members provided informed written consent to the implantation, according to procedures established by regulatory authorities.”

There were 12 (35.3%) articles in category 2c, which consists of descriptions of the caregiver’s role in the technical maintenance and upkeep of the BCI apparatus or in other procedural aspects of the study. One study in this category by Simeral et al. (2021), investigating the home use of a wireless BCI, comments on the disruption of wireless data acquisition as a consequence of caregiver intervention:

A review of the session logs found that the large majority of data disruptions (100 min) occurred when one or more caregivers were attending to T10 including rotating or shifting him in bed, suctioning, and other nursing care. During these periods, caregivers worked in close proximity to the bed including standing directly between the transmitters and antennas for several minutes at a time. Data were recorded but exhibited packet loss which was sometimes accompanied by substantial noise at the moment when the signal was recovered. In other cases, data flow stopped entirely when transmitters were removed during battery replacement, bathing and dressing.

Another example in this category mentions the role of the caregiver in setting up part of the BCI apparatus. Oxley et al. (2021) write, “System setup was performed by the caregiver with no expert knowledge, which involved attaching the receiver (ETU) to the chest with medical adhesive and launching the decoding software on Windows 10.” Still another article finds that their system requires less caregiver involvement: “Our study demonstrates that an intracortical LFP-based BCI can be used for independent communication without the need for recalibration, thereby reducing the need for caregiver and/or family intervention during communication” (Milekovic et al., 2018).

For category 2d, 9 (26.5%) of the 34 articles discussed ways in which the BCI enhanced communication and goal-directed behavior with the help of a caregiver. For instance, Fahimi Hnazaee et al. (2022) report, “[S]he used the UNP-BCI [Utrecht Neural Prosthesis-BCI] for caregiver calling and communication,” and Chaudhary et al. (2019) write that “…it was noteworthy that free voluntary spelling mainly concerned requests related to body position, health status, food, personal care and social activities suggesting that even with this slow speller the patient could relay his needs and desires to caretakers and family.” In a notable report, Milekovic et al. (2018) write that one of the sentences typed by a tetraplegic individual with their BCI was, “I want to thank all my caregivers who made the trip to Hawaii possible.”

Comments regarding caregivers were most common in studies that included participants with spinal cord injury (SCI) and ALS, while noting that SCI was the most commonly reported medical condition represented in the reviewed articles. Table 3 breaks down the comments regarding caregivers by category as a function of the reported medical condition of study participants. Comments regarding caregivers were also most common in studies involving BCIs for cursor control, while noting that cursor control was the most common function for BCIs in the studies included in this review. Table 4 gives the number of caregiver mentions by category according to BCI function. Analysis along these dimensions was limited by the fact that many of the studies that included heterogenous medical conditions, or that were studying multiple BCI functions within the same study, did not specify which participant-caregiver dyads were being commented on, or which BCI function was relevant to the comment of interest. Therefore, Table 3 likely over-counts the mentioning of caregivers by underlying medical condition and Table 4 by BCI function.

**Table 3:** Number of comments regarding caregivers by category as a function of the reported medical condition of study participants.

Medical condition	Number of comments regarding caregivers by category				
	1	2a	2b	2c	2d
Spinal cord injury	1(*)	11(**)	3(**)	5(**)	1(*)
ALS	1(*)	8(**)	2(**)	5(**)	6(**)
Stroke	3(*)	4(*)	3(***)	1(*)	1(*)
Locked-in syndrome		1	2	1	2
Upper extremity amputation					
Spinocerebellar degeneration					
Blindness		1			
Essential tremor					
Tetraplegia from unspecified cause		2			

See Appendix B for the categories of comments regarding caregivers. *Studies that include research participants with different underlying medical conditions and that do not identify which participants are referred to when caregivers are mentioned. For instance, Milekovic et al. (2018) write that BCIs will “provide greater and more extensive interactions with their friends, family, and caregivers,” which fits into category 2d. Since their trial included participants with brain stem stroke and others with ALS, one asterisk is added to the stroke row and another is added to the ALS row. An entry of “11(**)” in the spinal cord injury row and 2a category column signifies that there were eleven mentions of caregivers in trials that involved participants with spinal cord injuries. The two asterisks signify that two of the eleven mentions occurred in trials that had participants with different medical conditions, some of which were spinal cord injuries, but that did not specify the participant/caregiver being commented on.

**Table 4:** Number of caregiver mentions by category as a function of the BCI function.

BCI function	Number of comments regarding caregivers by category				
	1	2a	2b	2c	2d
Speech-related	1	5(*)	1	2	2
Cursor control	2(*)	14(**)	2(*)	7(*)	5
Limb movement	1(*)	2(*)	2(*)	1(*)	1
Sensory feedback					
Vision-related		1	1		
Motor imagery decoding only		2	1		
Flight simulator					

See Appendix B for the categories of comments regarding caregivers. *Studies that include BCIs with different functions. For instance, Rubin et al. (2023) investigate cursor control and control of a robotic and native arm. They also mention caregivers in category 1, 2a, 2b, and 2c. As a result, an asterisk was added to both the cursor control row and the limb movement row in the appropriate columns. An entry of “14(**)” in the cursor control row and the category 2a column signifies that there were fourteen mentions of caregivers in trials that studied BCIs to enable cursor control. The two asterisks in that entry signify that two of the fourteen mentions occurred in trials that investigated multiple BCI functions, one of which was cursor control.

We found a statistically significant difference in average word count per statement of interest by comment category (*F* = 6.657, *p* < 0.005). The length of the comments in categories 2b (“describing the caregiver’s role with regard to the consent process,” average word count (avg) = 39.8, standard deviation (stdev) = 42.1), 2c (“describing the caregiver’s role in the technical maintenance and upkeep of the BCI apparatus or in other procedural aspects of the study,” avg. = 46.4, stdev = 21.8), and 2d (“discussing the ways in which the BCI enhanced communication and goal-directed behavior through caregiver involvement,” avg. = 38.6, stdev = 17.9) were greater on average than those in categories 1 (“comments about the importance of caregivers in general,” avg. = 19.6, stdev = 8.9) and 2a (“thanking the caregivers involved in the study,” avg. = 14.6, stdev = 6.08). However, a post-hoc Tukey test revealed that there was a statistically significant difference in the length of the statements of interest only between categories 2a and 2b (q level = 6.485) and between categories 2a and 2c (q level = 4.45006). The q levels for the other pairwise comparisons were all below the critical level of 4.008, which is the critical q level for a 5% significant level, 5 groups, and a degree of freedom of 47 (given by the ANOVA) (Elvers, 2020).

## 4 Discussion

The conditions that qualify individuals for inclusion in neural device trials (e.g., quadriplegia from stroke or spinal cord injury) often lead to their reliance on others for everyday activities and functions, such as bathing, feeding, and toileting. Some also face social marginalization due both to ableism and to physical obstacles in engaging with others. For these individuals, caregivers often play a centrally important role in their lives and in enabling and supporting their activities and interests. The vital support that caregivers provide also extends to the realm of clinical research trials, and while this has been increasingly recognized in certain areas of neuroscience such as in clinical trials for Alzheimers, less scholarly work has been undertaken to investigate their specific roles in BCI research.

One way to expand our understanding of the extent of caregiver involvement in clinical BCI research is to review the published BCI literature and analyze the different ways that caregivers are mentioned. In this review, we found that nearly half of the studies of implantable BCIs include at least one mention regarding caregivers. To better understand the variety of ways in which caregivers are mentioned, we cataloged them first into general comments into two groups: comments about caregivers in general and observations about caregivers in the trial in particular. This last grouping was further divided into four categories to better understand the different ways in which caregivers were discussed in the literature. The largest category, representing the most common way of mentioning caregivers, consists of direct statements thanking them for their participation in the study, most often in the acknowledgement section of the article. Involvement in research places a burden on caregivers, which often goes under-appreciated (Cameron et al., 2020). Just as participants make time in their schedules to participate in trials, many caregivers make a substantial effort to help participate in research. Though this category of caregiver discussion by itself provides limited insight into the specific nature of the caregiver’s participation, acknowledging caregivers in this way can serve to recognize and honor the demands and burdens that they shoulder in the research process.

The remaining categories, however, offer more detailed evidence in support of the wide-ranging role that caregivers can play in BCI research. One important issue relates to the consent process, and several articles comment on the role of caregivers in decision-making and consent. The fact that the issue of consent emerges in this review as a distinct category is perhaps not surprising, as many of the study participants in this research have communication-limiting conditions, such as anarthria from stroke or ALS, making efficient and reliable communication challenging. In these cases, caregivers can often help interpret the wants and needs of the individual. These findings provide further support and context for the recommendations offered by Vansteensel et al. (2022) on the importance of caregivers to the consent process.

More fundamentally, the fact that caregivers are important to decision-making reflects the relational nature of the participant’s autonomy and identity. There are two ways that relationality is understood (Nelson and Nelson, 1995; Ho, 2020). First, individuals rely on others causally to engage with the world, for the satisfaction of fundamental needs, and for the enactment of goals. In the case of BCIs for ALS, it might be causal assistance in carrying out acts of communication. In other cases, e.g., for those with profound motor disorders, participants rely on caregivers for basic everyday needs such as feeding and mobility. Relational autonomy puts emphasis on the causal dependence that we all have, but that dependence is often more pronounced for individuals with conditions such as ALS. Second, relationality is constitutive, where values, preferences, and goals are partially formed through the influence of those close to us, such as family members, close friends, and caregivers. As a result, researchers often have non-instrumental responsibilities to caregivers and family members that might be either role-based or relationship-based (Olson, 2019).

Another important category of caregiver discussion in this review, beyond involvement in decision-making and enhanced communication, involves the ways in which caregivers interact with the technical aspects of BCI upkeep and maintenance. This has great practical import to the successful development and implementation of BCI systems for home use, for instance, which is the primary focus of the methodological recommendations by Vansteensel et al. (2022). What our review reinforces is the importance of caregivers to the long-term care of implanted neural devices. For instance, caregivers might be responsible for the home care and day-to-day monitoring of implanted pedicles (Rubin et al., 2023); as a result, their participation with that regard is an important element to account for. Furthermore, for devices meant for home use, the design of a BCI system should take into account the physical presence of a caregiver that might degrade signal transmission between implanted device and receiver (Simeral et al., 2021). Since caregivers often play a central role in the lives of these individuals, they should likewise play a central role in clinical trials, especially when the trials investigate the use of neural devices outside the confines of a laboratory.

Caregivers can be important for different reasons. One is recognizing that they provide vital support to both the research participant and to researchers. Caregivers may provide logistical support, helping with scheduling sessions, recovering from surgery, and ensuring efficient transportation to and from research sites. They also can provide emotional support in showing care and affection when needed, and sustaining the participant’s motivational state in respect to the clinical trial. Finally, caregivers act as expert knowers who can share a familiar third-person perspective on how the participant is doing, providing information that neither the participant nor the researcher can obtain by themselves. Our review provides evidence how these different forms of support manifest in BCI trials and provide some insight into their importance for the trial’s success. It is interesting to note that the one of the trials with the most detailed discussion of caregivers was the interim safety report on the Braingate interface system (Rubin et al., 2023). We understand this fact as a reflection of the importance of caregivers to both the research itself and to the participants, and we believe the discussion of the caregiver role should not be limited to safety reports but should be more systematically included in BCI trials generally.

There are several limitations to our review. First, our query terms were chosen to focus on paralysis-, speech-, and vision-related BCI research, which currently represent the major experimental applications of the technology, and as a result, studies for other conditions, where caregiver involvement could conceivably be different, are likely under-represented. For instance, studies on BCIs for neuropsychiatric diseases, such as depression, were not included in this review (Oganesian and Shanechi, 2024). Second, our screening process might have excluded studies involving non-implantable BCIs that could have provided insight into how caregivers are involved in BCI research (see Jadavji et al., 2022 for an example of a non-implantable BCI study, and see Abiri et al., 2019 for a review of non-implantable BCI studies) We chose to only include implantable devices, because the demands of surgery and post-operative follow-up as well as the longevity of these studies represent cases in which the need for caregiver support might be most pressing and demanding.

Nonetheless, a promising area for future research would be to compare the role of caregivers in non-implantable versus implantable BCI trials (see Lebedev and Nicolelis, 2017 for a summary of different methods of signal sampling). It is possible that some patterns of care that caregivers provide in non-implantable BCI trials would be qualitatively similar to those in implantable trials, and some might be significantly different. For instance, it might be the case that helping participants get to and from frequent study visits or providing emotional support through a long trial might be similar across both kinds of trials. Whereas it might be the case that caregivers of those in implantable device trials would have to look out for scalp infections near the surgical implantation site (or monitor for other kinds of medical comorbidities), even weeks or months after surgery. On the other hand, caregivers of participants in non-implantable device trials, for instance those with devices used in part at home, might have responsibility for device setup and calibration in the home (e.g., helping put the device on, making sure the leads are making sufficient contact with the skin, etc.). Which of these kinds of support are more demanding (or rewarding) and how researchers should be attentive to this care deserves additional study.

Third, whether or not caregivers are mentioned in published material may not accurately reflect the actual extent to which they were involved in the research. This limits the ability to make systematic comments about how caregivers are involved in BCI trials. For example, not mentioning caregivers in an article does not mean that they were not involved, and when they were mentioned, the extent of their involvement is not always detailed. Nevertheless, the lack of mention of caregivers in the majority of articles in this review is itself a notable finding.

In an effort to systematically address and acknowledge caregiver burden, responsibility, and support, we support the implementation of a standard by which caregivers are more formally incorporated into clinical trials. This might require further research on the roles that caregivers already have within clinical trials to ensure that they are sufficiently acknowledged and included in study designs. Formal protocols will likely include inclusion and exclusion criteria on the basis of need for caregiving support throughout the trial duration.
