# Supplementary material for: Caregivers in implantable brain-computer interface research: a scoping review
Source: Front Hum Neurosci. 2024 Oct 31;18:1490066. doi: 10.3389/fnhum.2024.1490066 (PMC11560881; doi:10.3389/fnhum.2024.1490066)
Supplement: Supplementary file 1 [file Table_1.docx]

| **Categories** | **Examples** |
| --- | --- |
| 1. Comments about the importance of caregivers in general | - "Anarthria hinders communication with family, friends, and caregivers, thereby reducing patient-reported quality of life” (Moses et al., 2021). - "Most individuals with tetraplegia depend on caregivers for mobility and physical interaction with their environment” (Simeral et al., 2011). - "People with quadriparesis may have greater contact with caregivers attending to health-related needs than those with epilepsy or Parkinson disease; consequently, comparisons with devices intended for ambulatory populations are imperfect” (Rubin et al., 2023). |
| 2a. Thanking the caregivers involved in the study | - "We thank participant T12 and her caregivers for their generously volunteered time and effort as part of the BrainGate2 pilot clinical trial” (Willett et al., 2023). - "We thank participant T5 and his caregivers for their dedicated contributions to this research” (Willett et al., 2021). - "We thank.... the participant’s caregivers for logistic support” (Moses et al., 2021) - "We thank participants T5, T11 and their caregivers for their generously volunteered time and effort as part of our BrainGate2 pilot clinical trial” (Wilson et al., 2020). - "We thank participants T10, T5 and their families and caregivers” (Simeral et al., 2021). - "The authors would like to thank participants T6, T5, T7, and their families and caregivers…” (Pandarinath et al., 2017). - "We thank participant T5 and his caregivers for their dedicated contributions to this research” (Stavisky et al., 2017). - "We thank the patient and his family” (Chaudhary et al., 2022). - "The authors would like to thank Participant T9 and his family” (Jarosiewicz et al., 2016). - "We thank our study participants for their commitment and effort for this study, and we also give our special thanks to the families and the caregivers of our study participants for their support to the participants” (Degenhart et al., 2018). - "We thank the BrainGate participants and their families for their contributions to this research” (Rastogi et al., 2021). - "We thank... the caregiver staff at the Boston Home for their contributions to this research” (Masse et al., 2014). - "We are grateful to the patients who participated in this study and their caregivers” (Mitchell et al., 2022). - "We would like to thank B G and her husband for their extraordinary commitment to this study” (Grani et al., 2022). - "The investigators thank the participants, their families, and caregivers for their extraordinary dedication to this research” (Rubin et al., 2023). - "The authors would like to thank participants T5, T8, T10, and their families” (Brandman et al., 2018). |
|  | - "The authors would like to thank participants T5, T6, and their families and caregivers” (Even-Chen et al., 2017). - "We thank participants S3, T2, T6, T7, and their families” (Jarosiewicz et al., 2013). - "We thank participants T5, T6, T7, T8 and their caregivers and families” (Willett et al., 2019). - "The authors would like to thank participants S3 and T1 and their families” (Jarosiewicz et al., 2013). - "The authors would like to thank participant T10 and his family” (Hosman et al., 2021). - "We thank the study participant and his family for their dedication and support” (Skomrock et al., 2018). |
| 2b. Describing the caregiver’s role with regard to the consent process | - "Informed consent was obtained from FL and his family for this implantable BCI study aiming to achieve real-time neural signal decoding and subsequent control of a high-performance prosthetic limb” (Jiang et al., 2022). - "The legally responsible family members provided informed written consent to the implantation, according to procedures established by regulatory authorities." "The patient was visited at home by authors HT and JL, and thorough discussions were held with the legally responsible family members (wife and sister) in order to establish convincing evidence of the patient’s informed consent and firm wish to undergo the procedure. The legally responsible family members then provided informed written permission to the implantation and the use of photographs, videos, and portions of his protected health information to be published for scientific and educational purposes. In addition, a family judge at the Ebersberg county court gave the permission to proceed with the implantation after reviewing the documented consent and a visit to the patient” (Chaudhary et al., 2022). - "All participants or their guardians gave written informed consent to participate in the study, which was approved by the ethics committee of Osaka University Hospital” (Shiraishi et al. 2020). - "Consent was further observed and attested to by a family member and third party witness” (Milekovic et al., 2018). - "Informed consent was obtained by the participant and his legal guardian for all procedures and experimental studies” (Brumberg et al., 2011). - "Any implanted medical device must have an adequate safety profile before clinical use so that patients, caregivers, and healthcare professionals can make informed decisions about risks and benefits” (Rubin et al., 2023).* |
| 2c. Describing the caregiver’s role in the technical maintenance and upkeep of the BCI apparatus or in other procedural aspects of the study | - “…[T]he large majority of data disruptions… occurred when one or more caregivers were attending to T10 including rotating or shifting him in bed, suctioning, and other nursing care. During these periods, caregivers worked in close proximity to the bed including standing directly between the transmitters and antennas for several minutes at a time. Data were recorded but exhibited packet loss which was sometimes accompanied by substantial noise at the moment when the signal was recovered. In other cases, data flow stopped entirely when transmitters were removed during battery replacement, bathing and dressing” (Simeral et al., 2021). - "Inclusion in the study required a caregiver willing to participate with study procedures” (Mitchell et al., 2022). - "In many cases, these [adverse events] were caused by overly enthusiastic preventive care of the pedestal site by a caretaker or family member, and these resolved after reeducating caregivers." "In a second participant during this early period, caregivers inadvertently applied a sudden axial force to the participant's head and pedestal while placing the participant back into bed” (Rubin et al., 2023). - "...[A]dditional clinical and experimental imperatives include the following: minimizing the amount of user effort required to engage the system; enhancing user engagement during early use; eliminating calibration procedures that provide no feedback (i.e. an explicit open-loop imagery step) for users with impaired levels of alertness; early confirmation to users and caregivers that the BCI is working; and, a dramatic increase in research efficiency” (Brandman et al., 2018).* - "Following the completion of a blockset, participants were given as long a break (to request a drink from a caregiver, etc.) as desired before starting the subsequent blockset” (Pandarinath et al., 2017). - "A future self-calibrating, fully implanted wireless system could in principle be used without caregiver assistance, would have no cosmetic impact, and could be used around the clock” (Pandarinath et al., 2017).* - "They used an LFP-based BCI with a decoder unchanged for 76 and 138 days to communicate with family members, type messages, and send emails, without a loss of performance. Our study demonstrates that an intracortical LFP-based BCI can be used for independent communication without the need for recalibration, thereby reducing the need for caregiver and/or family intervention during communication” (Milekovic et al., 2018). - "Further iterations of the iBCI software suite are required to shift to a user-centric design and allow for independent use of the system by iBCI users and caregivers” (Weiss et al., 2019).* - "With help of a caregiver, patients were able to voluntarily use the system at home without any intervention from the research team after session 12 for patient 1 and after session 2 for all others.” |
|  | - "...[T]he current neurofeedback based BCI system has several limitations, as several software and hardware modifications would need to be implemented before the system could be used independently by the family or caretakers without technical oversight. The BCI-software is presently being modified to improve communication quality and rate and the self-reliance of the family” (Chaudhary et al., 2022). - "Implantable BCIs have enabled increasingly sophisticated functionality to users, but it is challenging to maintain high performance over long periods of time without the need to retrain or recalibrate, which can be time-consuming and require intervention by a research team or a caregiver” (Luo et al., 2023).* - "System setup was performed by the caregiver with no expert knowledge, which involved attaching the receiver (ETU) to the chest with medical adhesive and launching the decoding software on Windows 10." "While scalp EEG systems can achieve single binary click selection, the current endovascular approach overcomes the problem of complex daily caregiver-dependent electrode setup” (Oxley et al., 2021). |
| 2d. Discussing the ways in which the BCI enhanced communication and goal-directed behavior through caregiver involvement | - "She used the UNP-BCI [Utrecht Neural Prosthesis-BCI] for caregiver calling and communication." "UNP5 is a man who was diagnosed with ALS in 2011. He is quadriplegic and anarthric, receives tracheostomy invasive ventilation, and uses an eye gaze device for communication with his family and caregivers, as well as small movements of the jaw to answer closed questions” (Fahimi et al., 2022). - "...[I]t was noteworthy that free voluntary spelling mainly concerned requests related to body position, health status, food, personal care and social activities suggesting that even with this slow speller the patient could relay his needs and desires to caretakers and family." "Intelligible phrases may contain words with spelling mistakes or incomplete words, but the family or experimenter identified and agreed upon their meaning” (Chaudhary et al., 2022). - "T5 used the tablet [for typing] to send text messages to friends, family, and the research staff through the Google Voice program." "T5 enjoyed messaging friends and family and watching videos, sending his first text messages ever via the iBCI in this study” (Nuyujukian et al., 2018). - An example of a sentence typed by study participants using the LFP-based BCI: "I want to thank all my caregivers who made the trip to hawaii possible” (Milekovic et al., 2018). - "This study is a step toward a reliable and robust BCI that will allow people with LIS to communicate independently and, therefore, provide greater and more extensive interactions with their friends, family, and caregivers” (Milekovic et al., 2018).* - "Two participants with flaccid upper limb paralysis due to ALS and dependent on caregivers used the ambulatory motor neuroprosthesis in conjunction with eye-tracking to control Windows 10 and independently conduct remote communication, online shopping and banking tasks” (Oxley et al., 2021). |
|  | - "Patients also used the system to express care needs to the caregiver” (Mitchell et al., 2023). - "She used the system whenever she went outside, where lighting conditions made eye tracking impossible; in those circumstances, she relied on the system as the only means of communication and also used it to draw the attention of her caregiver by using the brain click to select a button that generated a sound” (Vansteensel et al., 2016). - ​​"Individuals such as these [with locked-in syndrome] may use augmentative and alternative technologies (AAT) to select among options on a communication board, but this communication can be slow, effortful, and may require caregiver intervention” (Angrick et al., 2024). |

### **Appendix B**: Categories and examples of comments regarding caregivers in iBCI studies

See Appendix A for references.

*These statements are comments about caregivers in general, not about specific observations from the research trial, but that belong thematically to the subcategories.
